# Difficult-to-culture micro-organisms specifically isolated using the liquid-liquid co-culture method – towards the identification of bacterial species and metabolites supporting their growth

**DOI:** 10.1099/mic.0.001581

**Published:** 2025-07-08

**Authors:** Atsushi Hisatomi, Takanobu Yoshida, Tomohisa Hasunuma, Moriya Ohkuma, Mitsuo Sakamoto

**Affiliations:** 1 Microbe Division/Japan Collection of Microorganisms, RIKEN BioResource Research Center, Tsukuba, Ibaraki 305-0074, Japan; 2 Engineering Biology Research Center, Kobe University, Kobe 657-8501, Nada, Japan; 3 Graduate School of Science, Technology and Innovation, Kobe University, Kobe 657-8501, Nada, Japan; 4 NODAI Culture Collection Center, Tokyo NODAI Research Institute, Tokyo University of Agriculture, Setagaya-ku, Tokyo 156-8502, Japan

**Keywords:** co-culture, human gut, isolation method, metabolomic analysis, symbiotic relationship, *Waltera intestinalis*

## Abstract

In this study, the liquid-liquid co-culture method was applied using faecal samples and specific bacterial species as growth-supporting bacteria. We aimed to isolate new, difficult-to-culture bacterial species using metabolites produced by supportive bacteria to promote the growth of small bacteria selected using filter treatment. This study aimed to identify the supporting bacteria and their metabolites that promote the growth of these isolates. Analysis of the 16S rRNA gene sequences of the isolates obtained by co-culture revealed that they were *Waltera* spp., *Roseburia* spp. and *Phascolarctobacterium faecium*. *Roseburia* spp. and *Waltera* spp. were isolated from several faecal samples, suggesting that they were specifically isolated using this culture method. We focused on *Waltera* spp. isolated from several faecal samples with unique shapes, from long to short or thin cells. The growth of *Waltera* spp. was not promoted by co-culture on the agar medium, suggesting that growth was only promoted by liquid-liquid co-culture. The growth of the selected small-sized *Waltera* spp. was promoted by co-culture, whereas the growth of the unfiltered long-cell *Waltera* sp. strain was suppressed by co-culture. The selected small *Waltera* spp. did not grow when the supporting bacterial supernatant was added, suggesting that the supporting bacteria and *Waltera* spp. had a symbiotic relationship through the continuous exchange of metabolites. Co-cultured supporting bacteria (diluted faecal samples) with selected small-sized *Waltera* spp. were predominantly *Bacteroides thetaiotaomicron* and *Escherichia coli*, compared with monoculture diluted faecal samples. We further confirmed the growth of filtered *Waltera* spp. by co-culturing them with *B. thetaiotaomicron* and *E. coli*. Additionally, when *B. thetaiotaomicron* and *E. coli* were co-cultured with the selected small *Waltera* spp., some nutrients and metabolites were reduced. Decreased metabolites were added to the medium, and selected small-sized *Waltera* spp. were cultured, but *Waltera* spp. did not grow. Therefore, it was again strongly suggested that continuous co-culturing with the supporting bacteria was important for the growth of *Waltera* spp. The liquid-liquid co-culture method used in this study can be used to isolate new and unique bacterial species from any environment, not just the gut microbiome. Furthermore, this co-culture method helped identify supporting bacteria and understand metabolite variations.

## Graphical Abstract

Micro-organisms that are difficult to culture were isolated using liquid-liquid co-culture. *Waltera* spp. only formed colonies through continuous co-culture in liquid media in the presence of *Escherichia coli* and *Bacteroides* spp.

## Data availability

The GenBank/EMBL/DDBJ accession numbers for the 16S rRNA gene sequences of strains 18YCFAH0.3Co1, 19YCFAH0.3Co1, 19cm19_5, 23YCFAHCo1, 23YCFAHCo2, 23YCFAHCo3, 31YCFAHCo1, 17CBH1 and 17EGH20 are LC865666, LC865667, LC865668, LC865669, LC865670, LC865671, LC865672, LC877907 and LC877908, respectively.

## Introduction

**Fig. 1.**
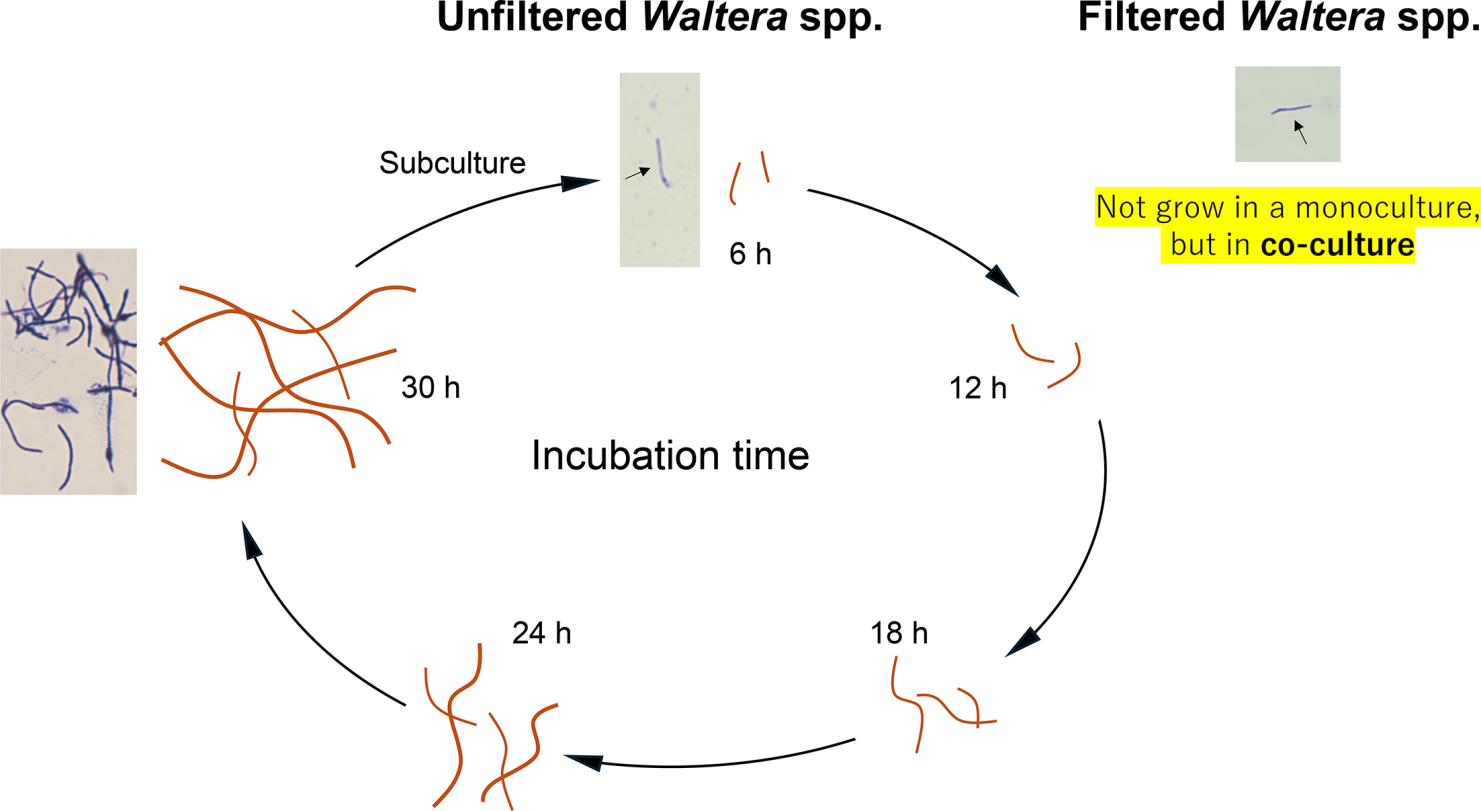
Observations over time of isolates cultured in liquid medium and image. The *Waltera* spp. cells elongated and proliferated over time, whereas filtered *Waltera* spp. grew only in the co-culture.

Recent health concerns have led to increased interest in gut bacteria. One topic of particular interest is the symbiotic relationship between bacteria, which is closely related to the pathogenicity, immunity and health of the human gut [1,3]. Therefore, understanding the key bacterial species and metabolites involved in symbiosis is important for understanding the development of gut bacteria. Moreover, isolation, cultivation and classification of human gut microbes are essential for industrial applications. It is known that 70–80% of gut microbes are uncultured [4]. Therefore, it is necessary to identify novel micro-organisms to develop new live biotherapeutic products. Recently, isolation techniques using co-cultures have been reported. Tanaka and Benno have reported a method for co-culturing difficult-to-cultivate micro-organisms by promoting their growth via metabolites produced by gut microbes by culturing faecal dilutions with a membrane filter that only permeates chemicals between agar media [5]. Ikeyama *et al*. have analysed the growth characteristics of succinate-utilizing *Phascolarctobacterium faecium* [6] isolated from humans and *Bacteroides thetaiotaomicron*, an intestinal commensal bacterium that produces succinate during co-culture, and have reported the promotion of growth by transferring succinic acid [7]. As mentioned above, various new species of bacteria and micro-organisms that are difficult to cultivate have been obtained using the co-culture method, and their cultivation mechanisms have been elucidated. Additionally, various co-culture methods have been used [8,10]; however, there are few cases where liquid-liquid co-culture methods using a liquid medium have been used for isolation. We obtained *Waltera* spp. via liquid-liquid co-cultivation. Moreover, we have reported their unique characteristics that *Waltera* spp. cells are small in size in the early stages of culture and elongate towards the later stages of culture; filtered *Waltera* spp. do not grow in monoculture but only in co-culture with supporting bacteria (SB) [11] (Fig. 1). However, it is unclear which bacterial species are involved in the growth of *Waltera* spp. and which metabolites are supplied in the co-culture. In this study, we continued to obtain difficult-to-culture micro-organisms using the liquid-liquid co-culture method and aimed to identify the bacterial species and metabolites that support the growth of isolates under co-culture.

## Methods

### Samples and equipment

**Fig. 2.**
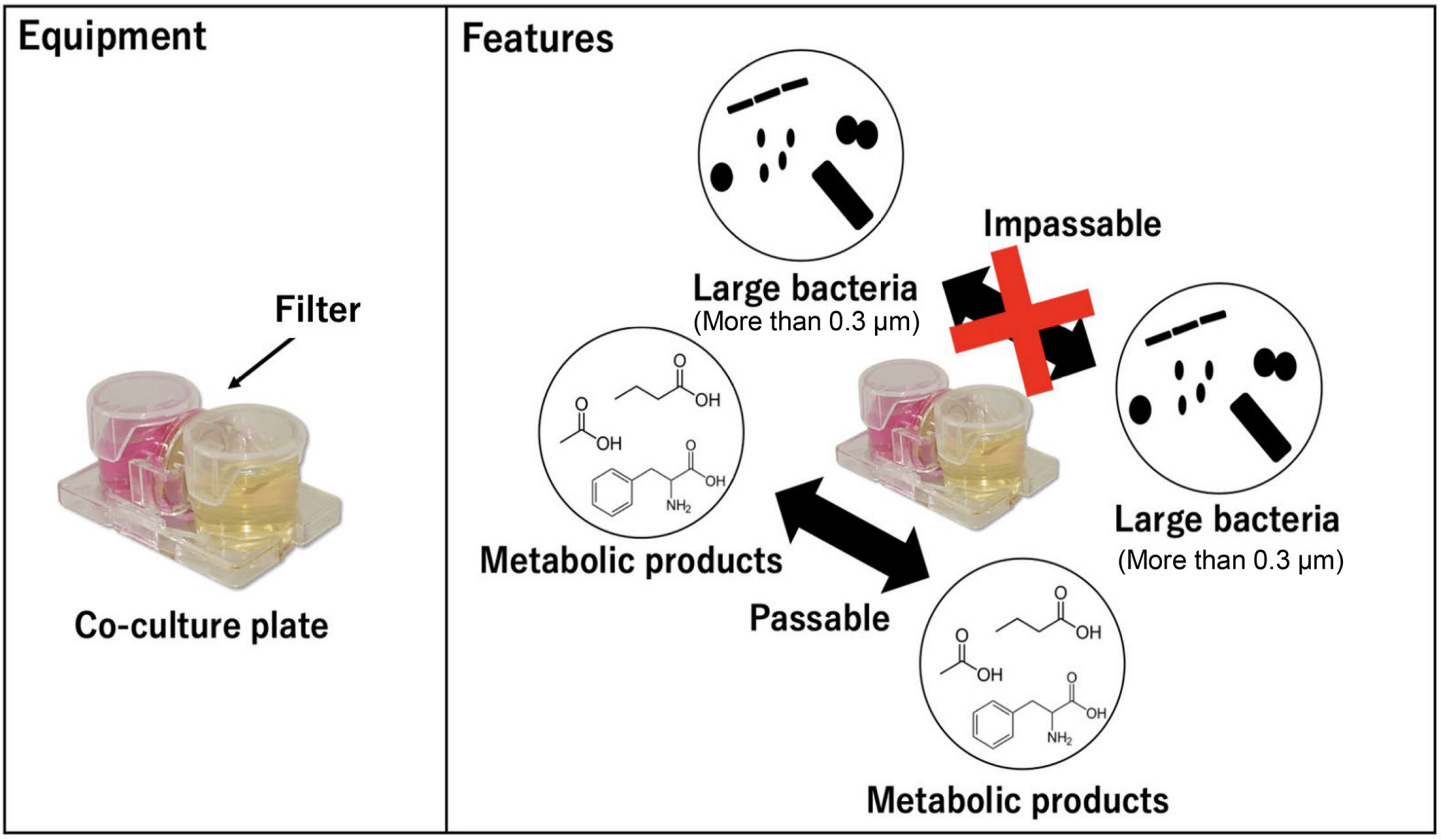
Co-culture equipment and features.

Faecal samples were obtained from healthy Japanese adults (no. 17: 30-year-old male, no. 18: 26-year-old female, no. 19: 28-year-old female, no. 23: 35-year-old male and no. 31: 82-year-old male) who provided informed consent. The faecal sample was suspended in 4.5 ml of PBS and degassed by flushing with nitrogen gas to remove oxygen. The sample (0.5 g) was diluted to 10^−3^. A horizontal co-culture vessel, UniWells^™^ Horizontal Co-Culture Plate (Fujifilm Wako Pure Chemicals), was used for culture as described above [11]. A membrane filter (0.1, 0.2 or 0.3 µm pore size) was inserted between the co-culture vessels (Fig. 2).

### Culture media and co-culture

**Fig. 3.**
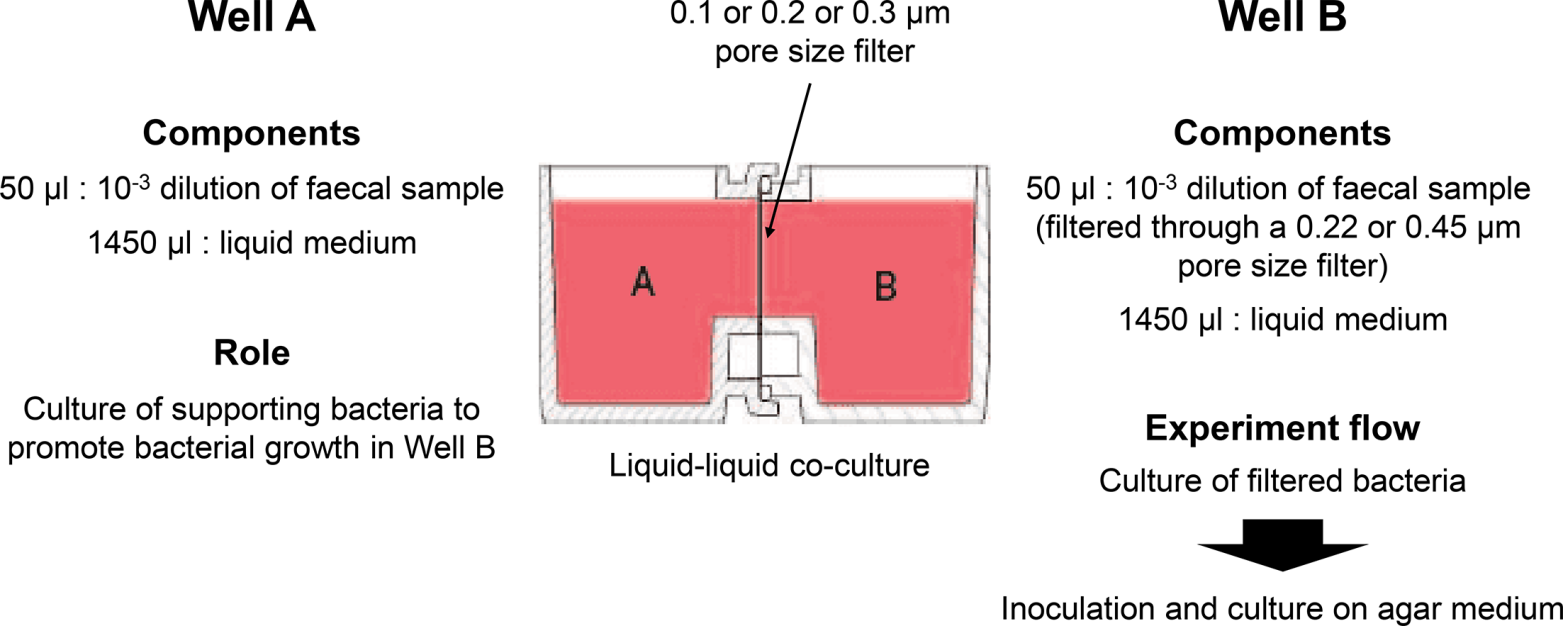
Overview of co-culture methods. Isolation was performed by co-culturing the supporting and filtered bacteria.

YCFA (JCM medium number 1130), mGAM (JCM medium number 1461) and Ruminococcus albus (JCM medium number 675) media were used. First, 1,450 µl of medium was added to each well. Then, 50 µl of the diluted faecal sample was inoculated into one side of the co-culture vessel as SB for growth. The other side was inoculated with 50 µl of a solution in which the main bacteria were removed by filtering the diluted faecal sample through a 0.45 or 0.22 µm pore size filter (defined as a filtered bacterial solution). A monoculture of 50 µl of filtered bacterial solution in 1,450 µl of YCFA liquid medium was also performed on one side of the co-cultures as a control. Cultivation in each medium was performed for 2 days with the above-mentioned co-culture vessels placed in an anaerobic chamber (Bactron 300) under 37 °C, H_2_/CO_2_/N_2_ (volume ratio: 0.5 : 0.5 : 9) conditions. One hundred microlitres of the co-culture or monoculture-filtered bacterial solution were inoculated onto various agar media and incubated for 2 days (Fig. 3). Thereafter, a 0.3 µm pore size filter was placed between the co-culture vessels, and samples were filtered through a 0.45 µm filter pore size for the experiment.

### Identification of isolates

The 16S rRNA gene was analysed using primers 27F and 1492R as described previously [12]. Sequencing was performed using SeqStudio (Thermo Fisher Scientific) according to standard methods and homology search in EzBioCloud [13].

### Co-culture of isolates on agar plate

SB were streaked onto half of the YCFA plate using an inoculation loop. *Waltera* spp. isolates filtered through a 0.45 µm pore size filter (defined as selected small-sized isolates) were similarly streaked on another half of the same YCFA plate. Each isolate was monocultured and used as the control. The effect of SB on the isolates was assessed by comparing colony formation and size [7].

### Growth of isolates

The selected small-sized isolates were cultured in YCFA medium for 48 h in monoculture and co-culture with the SB. Subsequently, the turbidity of the culture medium under each condition was measured at 660 nm using an Ultrospec 2100 *pro* spectrophotometre (Amersham Biosciences), and then, 100 µl of the culture was incubated on YCFA agar medium for 2 days.

### Supporting bacterial composition and metabolites

**Fig. 4.**
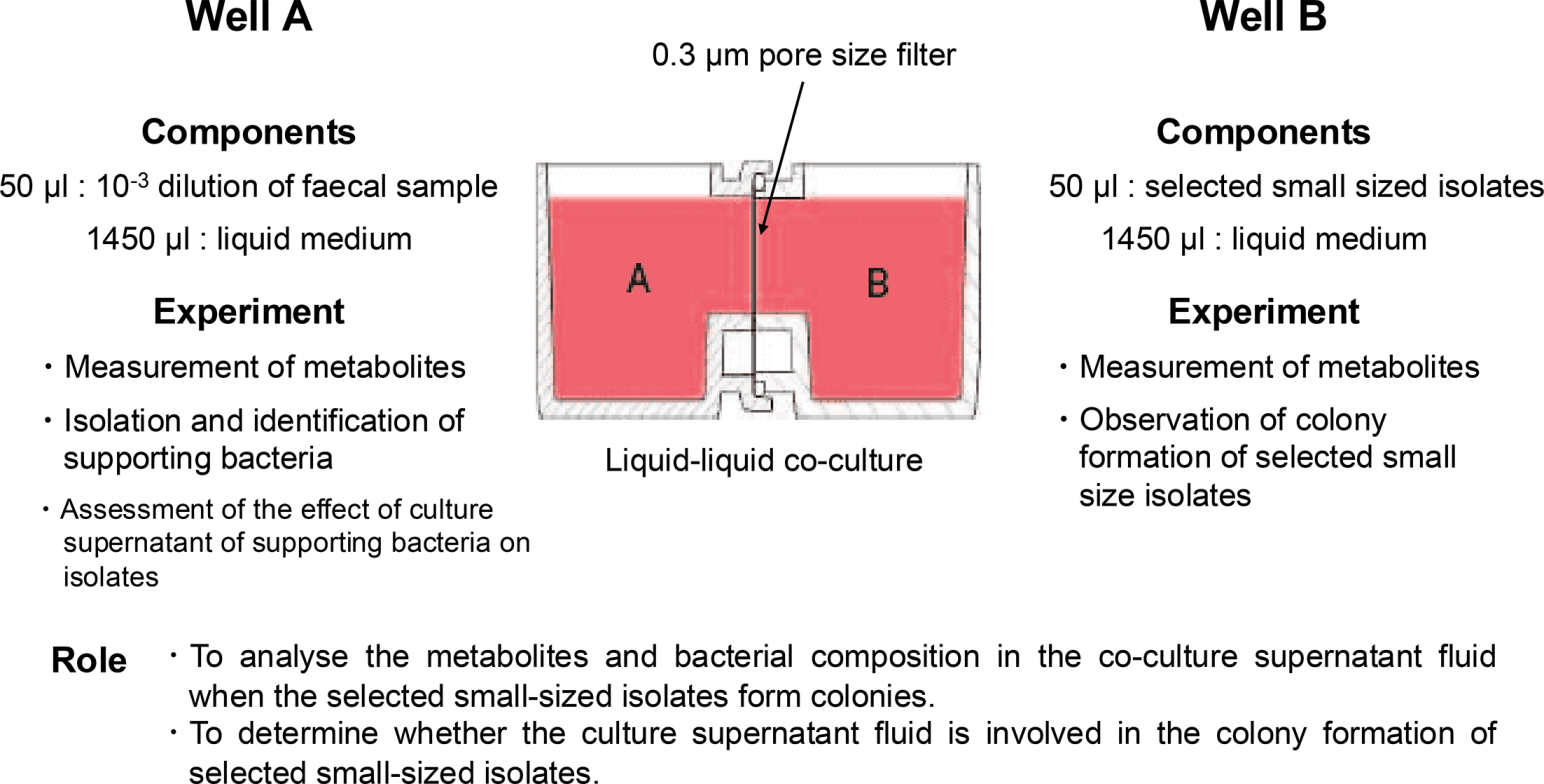
Overview of supporting bacterial composition and metabolites. The supporting bacterial composition and metabolites produced during colony formation of *Waltera* spp. isolates were analysed.

First, 1,450 µl of YCFA medium was added to each well. Then, 50 µl of SB and 50 µl of selected small-sized isolates were co-cultured for 12, 24, 36 and 48 h. Subsequently, 50 µl of selected small-sized isolates was also monocultured as a control. Next, 1.5 ml of a co-cultured solution was centrifuged (4,000 ***g*** for 10 min) and filtered through a 0.22 µm pore size filter to obtain the culture supernatant solution (defined as a co-SB supernatant solution) for each hour. Similarly, 1.5 ml of monocultured selected small-sized isolates was filtered every 12 h to obtain the culture supernatant solution (defined as monocultured isolate supernatant solution). Each culture was prepared for subsequent experiments (Fig. 4).

### Assessment of the effect of culture supernatant of SB on isolates

First, 1.5 ml of the co-SB supernatant solution of each culture time was added to one side of the co-culture vessel, and 50 µl of selected small-sized isolates to the other side (including 1,450 µl of YCFA medium) was co-cultured for 2 days. Subsequently, 100 µl of co-cultured solution, which added selected small-sized isolates, was cultured onto agar medium for 2 days.

### Measurement of SB metabolites

The preparation and analysis of the co-SB supernatant solution and the monoculture-isolated supernatant solution after 48 h were conducted as follows.

One millilitre of the culture was collected and centrifuged at 4,000 ***g*** at 4 °C for 10 min. Then, 450 µl of the supernatant was filtered to remove foreign materials using an Amicon Ultra-0.5 Centrifugal Filter Unit (3 KDa cut-off filter, Merck Millipore). The filtered supernatant was diluted 10-, 100- and 1,000-fold with 1 µM d-camphorsulfonic acid (as internal standard).

3-(4-Hydroxyphenyl)propionic acid was analysed in its derivatized form. Derivatization was performed using the LC (liquid chromatography)/MS/MS method package for short-chain fatty acids (Shimadzu), as previously described [14]. Briefly, the filtered supernatant was mixed with an equal volume of 400.8 µM 2-ethylbutyric acid (as the internal standard). The final concentration after the reaction is 10 µM. Equal volumes of 175 mM 3-nitrophenylhydrazine/75% methanol, 105 mM 1-ethyl-3-(3-(dimethylaminopropyl)carbodiimide)/75% methanol and 2.5% pyridine/75% methanol were mixed. The reaction proceeded at room temperature for 1 h. The reaction mixture was diluted fivefold with 0.5% formic acid/75% methanol.

LC/MS/MS analysis was performed under the following conditions.

Sugar phosphates and succinyl-CoA were analysed using a Mastro2 C18 column (2.0 mm×150 mm, particle size of 3 µm, Shimadzu GLC). MS conditions were as follows: mode of mass analysis, negative ion mode; nebulizer flow, 50 psi; dry gas flow rate, 10 l min^−1^; sheath gas flow rate, 12 l min^−1^; dry gas temperature, 300 °C; sheath gas temperature, 250 °C. The equipment and LC conditions were the same as those used in a previous study [15].

3-(4-Hydroxyphenyl)propionic acid was analysed using LC/MS/MS with a Nexera X3 high-performance LC system coupled to an LCMS-8060NX triple quadrupole mass spectrometer (Shimadzu).

The MS and LC conditions were determined using the method package for short-chain fatty acids (Shimadzu). The multiple reaction monitoring transitions were observed at 302.20>107.10 (+).

Other metabolites were analysed with the above-mentioned equipment.

The MS and LC conditions were identical to those described in the Method Package for Primary Metabolites Ver.3 (Shimadzu) and a previous report [16].

### Isolation and identification of SB

SB co-cultured for 24, 36 and 48 h were diluted to 10^−3^. One hundred microlitres of the diluted culture medium were incubated on agar medium for 2 days, and the resulting 48 colonies were identified using MALDI-TOF MS Microflex LT (Bruker).

### Co-culture with specific SB

We used *B. thetaiotaomicron* 17EGH20 (LC877908) and *Escherichia coli* 17CBH1 (LC877907) (these two strains are designated as BE), which were isolated from the SB. The isolates (17EGH20 and 17CBH1) and selected small-sized isolates were co-cultured in YCFA medium for 3, 6, 9, 12, 24, 36 and 48 h. Fifty microlitres of the selected small-sized isolates were also monocultured as controls. The co-cultured solution was centrifuged (4,000 ***g***, 10 min) and filtered through a 0.22 µm pore size filter to obtain a culture supernatant solution (defined as a co-BE supernatant solution) every hour. Then, 1.5 ml of the monocultured isolate supernatant solution was obtained every hour in the same manner. Each culture was used for subsequent experiments.

### Observation of colony formation of filtered isolates

The co-cultured solution (100 µl), which added selected small-sized isolates, was cultured onto an agar medium for 2 days.

### Assessment of the effect of culture supernatant of *B. thetaiotaomicron* and *E. coli* on isolates

First, 1.5 ml of co-BE supernatant solution for 12, 24, 36 and 48 h culture time was added to one side of the co-culture vessel, and 50 µl of selected small-sized isolates to the other side (including 1,450 µl of YCFA medium) was co-cultured for 2 days. One hundred microlitres of the co-culture solution containing the selected small-sized isolates were cultured on agar medium for 2 days.

### Measurement of BE metabolites

Preparation and analysis of the co-BE supernatant solution and the monocultured isolate supernatant solution at each culture time point were conducted as described above.

### Culture with metabolite added

Some of the metabolites whose concentrations increased in the early stages of co-culture and gradually decreased as detected by metabolomic analysis (Table S1, available in the online Supplementary Material) were added to YCFA liquid medium, and selected small-sized isolates were incubated for 2 days. Subsequently, 100 µl of the culture was incubated on YCFA agar medium for 2 days.

## Results

### Isolates

**Fig. 5.**
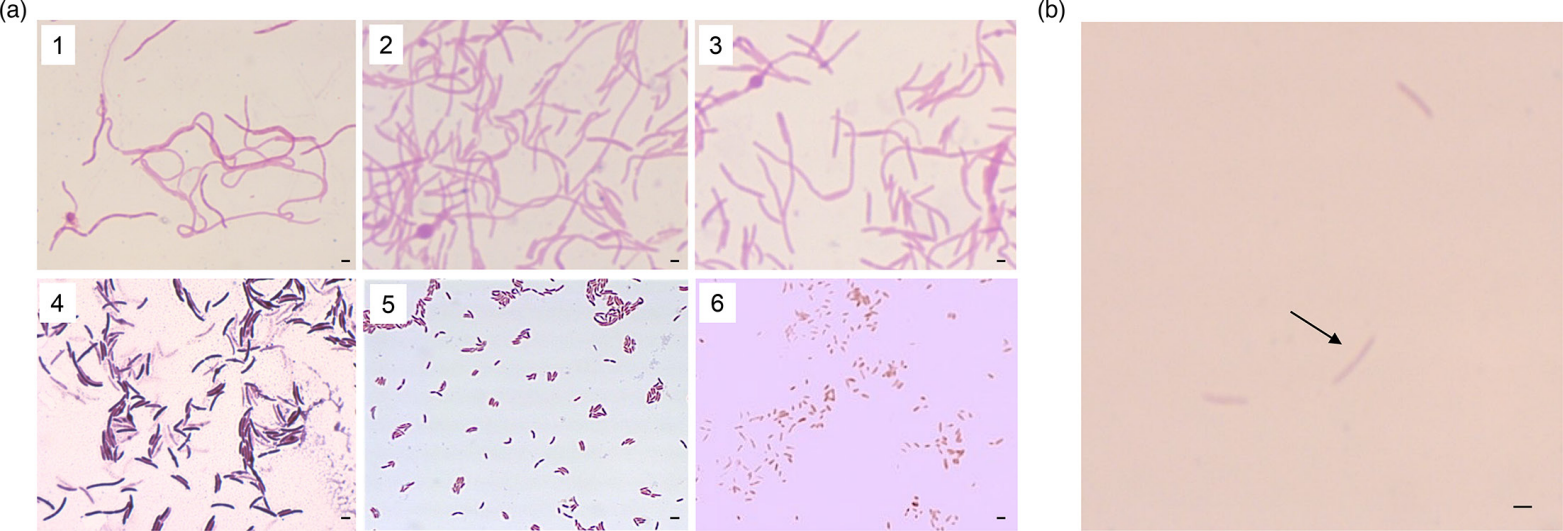
Observations of emerged cells. (**a)** Gram staining of strains isolated by co-cultures. The cells of strains 1–3 were long, wavy rods. The cell widths of strains 4–6 were smaller than those in the scale bars. Scale bars, 0.5 µm. Strains: 1, 17YCFAHCo10; 2, 18YCFAH0.3Co2; 3, 19YCFAH0.3Co2; 4, 19cm19_5; 5, 19YCFAH0.3Co1; 6, 18YCFAHCo1. (**b)** Gram staining of the strain 17YCFAHCo10 after treatment with a 0.45 µm pore size filter. Scale bar, 1 µm.

The presence of bacterial cells was microscopically confirmed in the extract of faecal samples filtered with a pore size of 0.45 µm, as described previously [11]. However, cells were not detected in extracts where samples were filtered through a 0.22 µm pore size filter. When samples filtered with a pore size of 0.45 µm were monocultured or co-cultured and the cultures were inoculated onto agar medium, no colonies formed from the monoculture medium. However, colonies formed on the agar medium in all faecal samples from the co-culture medium. Moreover, these bacteria were isolated only when the sample was filtered with a pore size of 0.45 µm, and a 0.30 µm pore size filter was placed between the co-cultures. In addition to the previously reported strains 17YCFAHCo10, 18YCFAH0.3Co2 and 19YCFAH0.3Co2 [11], the strains 18YCFAH0.3Co1, 19YCFAH0.3Co1, 19 cm19_5, 23YCFAHCo1, 23YCFAHCo2, 23YCFAHCo3 and 31YCFAHCo1 were isolated and designated based on their faecal sample numbers. As shown in 17YCFAHCo10, 18YCFAH0.3Co2 and 19YCFAH0.3Co2 [11], the cell diameters of 23YCFAHCo3 were 0.5–1.0 µm despite its filter treatment (Fig. 5a). These long-cell bacteria have also been isolated from YCFA, mGAM and Ruminococcus albus media. Furthermore, the cell diameters of these isolates extracted with a pore size of 0.45 µm were smaller than 0.45 µm (Fig. 5b). The cell diameter of the other strains was smaller than 0.45 µm (Fig. 5a).

### Sequences

16S rRNA gene sequence analysis showed that 17YCFAHCo10 and 18YCFAH0.3Co2 had 99.2% and 19YCFAH0.3Co2 had 100% nucleotide identity with *Waltera intestinalis* [11,17]. Whole-genome sequence analysis showed that 19YCFAH0.3Co2 belonged to *W. intestinalis*. 17YCFAHCo10 and 18YCFAH0.3Co2 were identified as *Waltera acetigignens* [11,17]. These three strains have been deposited in the Japan Collection of Microorganisms (JCM), RIKEN BioResource Research Center, under the accession numbers JCM 36211 (=19YCFAH0.3Co2), JCM 36210 (=17YCFAHCo10) and JCM 36378 (=18YCFAH0.3Co2), respectively. Other information on each sample and the similarity of each isolate to closely related species and accession numbers are given in the table (Table 1).

**Table 1.** Information on each sample and isolate

Faecal sample			Isolate			
No.	Sex	Age	Closest species	Homology (16S rRNA)	Strain	Accession number
17	Male	30	*W. acetigignens*	[11,17]	17YCFAHCo10	LC777694BTTZ00000000
18	Female	26	*P. faecium*	99.50%	18YCFAH0.3Co1	LC865666
			*W. acetigignens*	[11,17]	18YCFAH0.3Co2	LC781962BTUA00000000
19	Female	28	*Roseburia hominis*	100%	19YCFAH0.3Co1	LC865667
			*W. intestinalis*	[11,17]	19YCFAH0.3Co2	LC781963AP028995
			*Roseburia inulinivorans*	99.30%	19cm19_5	LC865668
23	Male	35	*R. hominis*	99.90%	23YCFAHCo1	LC865669
			*Roseburia difficilis*	99.70%	23YCFAHCo2	LC865670
			*W. intestinalis*	99.80%	23YCFAHCo3	LC865671
31	Male	82	*R. hominis*	100%	31YCFAHCo1	LC865672

### Growth of isolates on agar medium

**Fig. 6.**
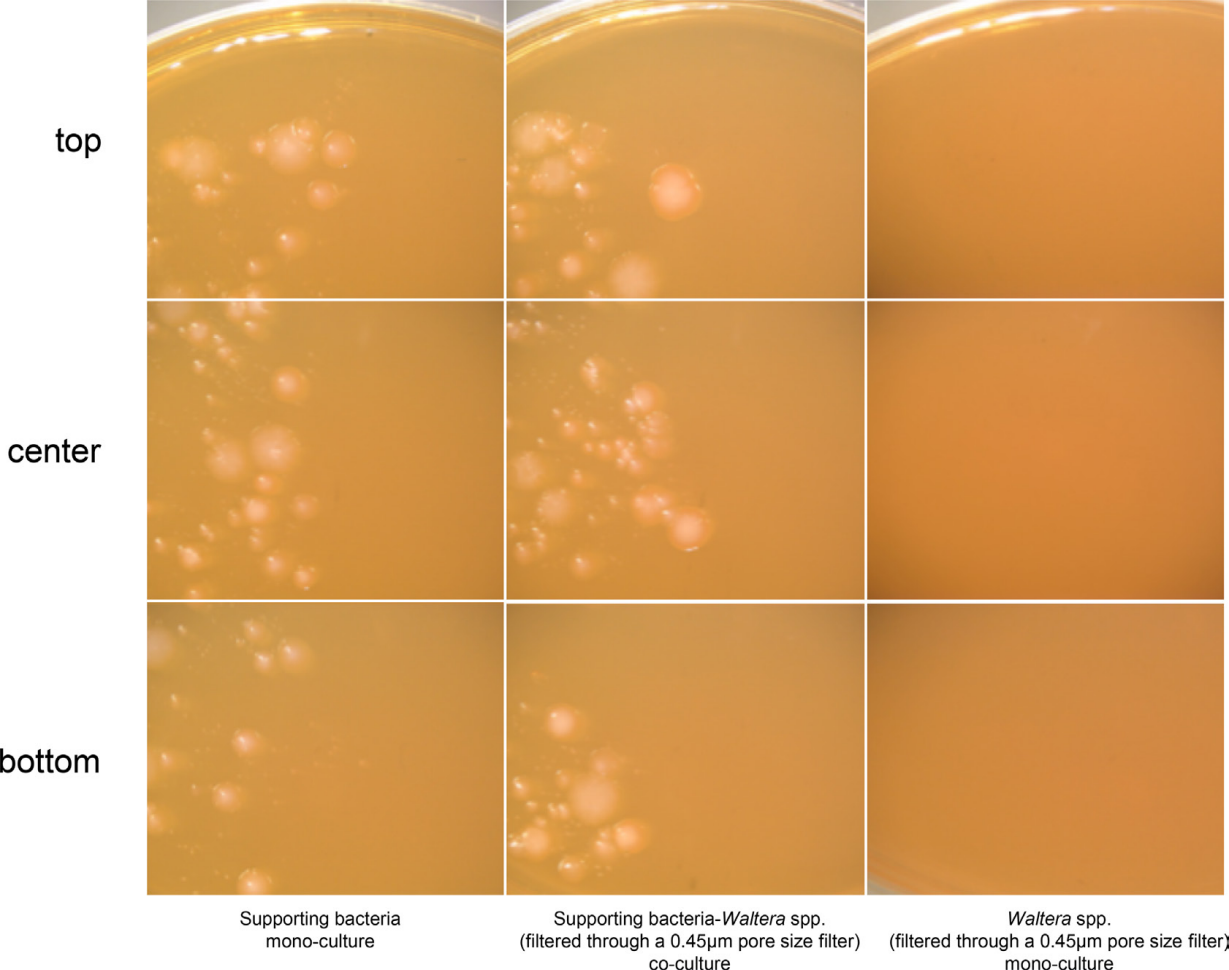
Growth stimulation of *Waltera* spp. isolate by SB. Strains were cultured in YCFA medium for 3 days at 37 °C in an atmosphere of 0.5 : 0.5 : 9 H_2_/CO_2_/N_2_.

The growth of *Waltera* spp. isolates on agar medium was compared between monocultures and co-cultures with SB. Colonies from both cultures were comparable in size (Fig. 6). Additionally, the selected small isolates did not form colonies under either of the conditions.

### Growth of isolates in liquid medium

**Fig. 7.**
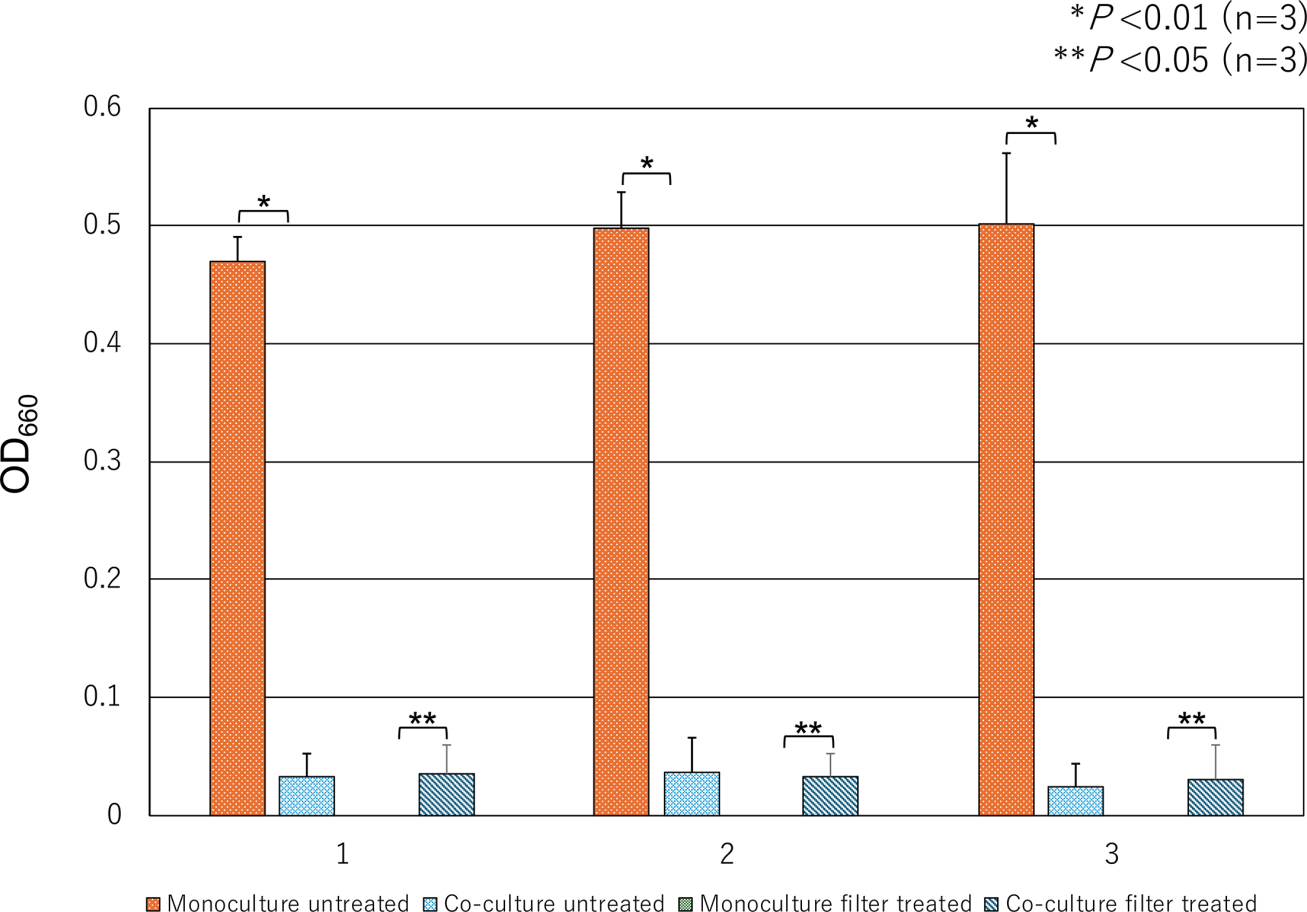
Comparison of turbidity in co-cultures and monocultures of 0.45-filtered and unfiltered isolates. Isolates filtered with a 0.45 µm pore diameter filter did not grow in monocultures but did grow in co-cultures. The unfiltered isolates grew better in monocultures than in co-cultures. These values indicate OD at 660 nm. Strains: 1, 17YCFAHCo10; 2, 18YCFAH0.3Co2; 3, 19YCFAH0.3Co2.

The turbidity of the *Waltera* spp. isolates was higher in the monoculture than in the co-culture under unfiltered conditions. However, in the cases of selected small-sized *Waltera* spp. isolates, an increase in turbidity was observed only during co-culture (Fig. 7). The growth of the *Waltera* spp. isolates was limited when co-cultured with or without treatment and was not as high as that of the isolates grown in monoculture without treatment. Additionally, unfiltered cells formed colonies when inoculated on agar medium after co-culture or monoculture, whereas selected small *Waltera* spp. formed colonies on agar medium only when co-cultured for 48 h. However, no colonies formed in co-cultures shorter than 48 h.

### Effect of SB

The selected small-sized *Waltera* spp. isolates were inoculated on one side of the co-cultivation unit, and the supernatant solution of the SBl culture incubated for 12, 24, 36 and 48 h was added to the other side of the co-cultivation unit for co-cultivation and then inoculated onto the agar medium; however, no growth or colony formation of the *Waltera* spp. isolates was observed. Additionally, the culture supernatant solution of the SB in the co-culture cup was replaced every 12 h to simulate co-culture and then inoculated onto agar medium; however, no growth or colony formation of the *Waltera* spp. isolates was observed.

### Metabolites of SB

Metabolomic analysis showed that indole, citrulline and 3-(4-hydroxyphenyl)propionic acid were detected only when the selected small-sized *Waltera* spp. isolates were grown in co-culture with SB for 48 h (Table 2).

**Table 2.** Metabolites under conditions that allow selected small-sized *Waltera* spp. isolates to form colonies Strains: 1, 18YCFAH0.3Co2; 2, 19YCFAH0.3Co2.

Metabolites (μM)	Co-culture		Monoculture	
	1	2	1	2
3-(4-Hydroxyphenyl)propionic acid	0.14	0.065	nd	nd
Citrulline	1.6	2.76		
Indole	326.60	282.13		

3-(4-Hydroxyphenyl)propionic acid, citrulline and indole were not present in the monoculture of selected small-sized *Waltera* spp. isolates but were present only after 48 h of co-culture.

nd, Not detected.

### Isolation of SB

Supporting bacterial colonies were isolated from the co-cultures. Different members of the constituent strains were compared with those of the monoculture (Table 3). Colonies of a limited number of species emerged from the co-cultured SB (diluted faecal samples), with the proportion of *E. coli* being high and *Bacteroides* spp. gradually increasing. However, colonies of *E. coli* were not predominant in the monoculture of the diluted faecal samples.

**Table 3.** Percentage of *E. coli* and *Bacteroides* spp. within SB (diluted faecal sample) under conditions where *Waltera* spp. isolates formed colony through co-culture

Incubation	Formed colony of SB each incubation time*E. coli* : *Bacteroides* spp. (%)		
	24 h	36 h	48 h
Monoculture	0 : 12.5	0 : 6.25	0 : 12.5
Co-culture with selected small-sized *Waltera* spp. isolates	25.0 : 4.17	45.8 : 4.17	41.7 : 14.6

The proportion of *E. coli* was high, and *Bacteroides* spp. gradually increased in the co-culture with SB (diluted faecal samples). The proportion of * E. coli* was low in the diluted faecal samples from the monoculture.

### Co-culture of isolates with *B. thetaiotaomicron* and *E. coli*

When selected, small-sized *Waltera* spp. isolates were co-cultured with *B. thetaiotaomicron* and *E. coli* for 48 h, *Waltera* spp. isolates grew and formed colonies on agar medium. However, no colonies formed in co-cultures shorter than 48 h. Additionally, no *Waltera* spp. isolates grew when the monoculture supernatants of the two SB were added to the selected small-sized *Waltera* spp. isolates and then cultured.

### Metabolites of *E. coli* and *B. thetaiotaomicron*

Metabolomic analysis showed that indole was only detected when selected small-sized *Waltera* spp. isolates were co-cultured with *E. coli* and *B. thetaiotaomicron* and increased proportionally with incubation time (Table 4). Metabolites, such as amino acids and organic acids, were identified for a 3 h culture time of co-cultivation but decreased in the later stages of cultivation (Tables5  6). Additionally, increases in metabolites, such as amino acids and organic acids, were observed (Table S2a, b).

**Table 4.** Indole (μM) detected when *W. acetigignens* 18YCFAH0.3Co2 and *W. intestinalis* 19YCFAH0.3Co2 were co-cultured with *B. thetaiotaomicron* 17EGH20 and *E. coli* 17CBH1 Strain: 1, 18YCFAH0.3Co2; 2, 19YCFAH0.3Co2.

Culture time (h)	Co-culture		Monoculture	
	1	2	1	2
0	nd	nd	nd	nd
3	7.14	11.10		
6	nd	nd		
9	nd	nd		
12	5.07	3.80		
24	45.95	62.16		
36	62.46	38.43		
48	76.94	126.62		

Indole was not detected in the monoculture of the selected small-sized *Waltera* spp. isolates, whereas in the co-culture, the amount detected increased with incubation time.

The higher the production, the more it is shown in red.

nd, Not detected.

**Table 5.** Metabolites (μM) decreased when *W. acetigignens* 18YCFAH0.3Co2 is co-cultured with *B. thetaiotaomicron* 17EGH20 and *E. coli* 17CBH1

Co-culture time (h)	Aspartic acid	Adenine	cAMP	Fumaric acid	Leucine	Maleic acid	Malic acid	Nicotinamide	Phenylalanine	Uracil
0	428.93	27.33	6.03	0.07	1,629.23	2.93	7.30	17.90	472.41	25.84
3	526.05	21.30	7.68	6.93	3,523.08	264.37	12.74	18.28	1,009.87	59.97
6	419.16	24.41	6.61	1.66	2,882.07	61.94	7.81	16.36	816.21	31.13
9	340.41	22.08	6.20	1.24	2,958.11	31.14	6.60	15.63	886.07	25.86
12	431.15	26.36	8.95	1.62	2,778.88	33.95	6.88	19.63	819.14	36.64
24	162.88	3.21	1.92	0.19	2,116.47	11.84	1.47	0.64	624.09	6.60
36	161.82	4.67	0.64	0.26	1,809.64	6.03	1.71	0.03	523.41	8.09
48	87.95	2.93	0.05	0.21	1,737.28	2.76	0.98	0.06	488.24	3.95

Some metabolites were detected in the early stages of co-culture and decreased during the later stages of culture.

The higher the production, the more it is shown in red.

**Table 6.** Metabolites (μM) decreased when *W. intestinalis* 19YCFAH0.3Co2 is co-cultured with *B. thetaiotaomicron* 17EGH20 and *E. coli* 17CBH1

Co-culture time (h)	Adenine	Aspartic acid	cAMP	Carnosine	Citric acid	Fumaric acid	Guanine	Leucine	Lysine	Maleic acid	Malic acid	Nicotina-mide	Thymine	Uracil
0	0.21	75.74	0.08	1.54	6.79	0.23	0.00	1,857.51	778.57	2.25	0.25	0.07	2.68	1.21
3	26.49	456.94	6.80	14.69	56.91	5.77	33.33	3,378.22	2,399.55	209.79	13.79	22.19	9.26	50.16
6	31.72	434.99	6.62	17.27	58.09	1.15	19.47	3,051.62	1,859.96	33.83	10.37	22.42	4.32	25.76
9	19.80	384.72	6.47	14.58	42.05	0.92	13.25	3,144.97	1,944.54	34.09	6.61	15.41	4.56	27.45
12	16.78	364.03	7.62	14.68	38.78	1.36	14.76	2,447.00	1,789.70	47.75	6.00	12.62	5.49	30.67
24	8.96	185.21	1.64	4.25	19.81	0.47	6.53	2,227.21	946.60	10.37	1.94	1.46	6.07	8.57
36	4.61	118.18	0.35	3.05	4.90	0.08	3.31	1,975.45	479.31	3.90	1.17	0.10	3.39	6.62
48	0.21	75.74	0.08	1.54	6.79	0.23	0.00	1,857.51	778.57	2.25	0.25	0.07	2.68	1.21

Some metabolites were detected in the early stages of co-culture and decreased during the later stages of culture.

The higher the production, the more it is shown in red.

### Effects of metabolites

The selected small-sized *Waltera* spp. isolates were cultured by adding each compound to the YCFA medium with reference to the metabolites whose concentrations increased and gradually decreased in the early stages (3 h) of co-cultivation as detected using metabolomic analysis. However, growth was not enhanced, and no colonies formed on the agar medium.

## Discussion

In this study, a liquid-liquid co-culture method was employed to isolate unique micro-organisms. This method is still minor and is expected to be the method for isolating new and unique bacterial species in the future. The filter pore size used for the filtration of faecal dilutions and the filter pore size inserted between the co-cultivators were investigated to isolate the bacteria. Bacteria were only isolated when using a 0.45 µm pore size filter for the faecal dilutions and a 0.3 µm pore size filter inserted between the co-culture vessel. The genera *Waltera*, *Roseburia* and *Phascolarctobacterium*, which are known to be difficult-to-culture micro-organisms, were successfully isolated. Similar to previous studies [11], *W. intestinalis* was isolated in the present study. *W. intestinalis* has only been isolated from pigs [18], and this is the first method used to isolate it from the human gut [11]. Additionally, *W. acetigignens*, which was isolated as a new species, is closely associated with *W. intestinalis*. In particular, *Waltera* spp. were isolated from several samples and all media used, suggesting that they were specifically acquired by the co-culture method used in this study. *W. intestinalis* possesses genes associated with the biosynthesis of Ruminococcin A (RumA), which is highly active against pathogenic *Clostridium* spp. [19]. The low production yield of *Ruminococcus gnavus* (<1 µg l^−1^) and the difficulty of culturing it make high-quality production difficult [20], suggesting that the *Waltera* spp. could be used as an alternative production source of RumA [21]. As *P. faecium* is known to be supported by succinate [7], it is suggested that growth was also supported by the liquid-liquid co-culture in this study. *P. faecium* can produce short-chain fatty acids and can be associated with the metabolic state of the host [22]. Furthermore, *P. faecium* has been reported to improve obesity and metabolic disorders in mice and has potential applications in the development of disease-related drugs [23]. As strains of *Roseburia* spp. were isolated from several samples, as well as bacteria of *Waltera* spp., it is expected that this method can be used to specifically obtain *Roseburia* spp. This culture method is expected to make a significant contribution to the acquisition of *Roseburia* spp. There is evidence that *Roseburia* spp. have a positive impact on several diseases, including inflammatory bowel disease, atherosclerosis, alcoholic fatty liver, colorectal cancer and metabolic syndrome, and are expected to be a non-toxic next-generation probiotic [24,25]. As described above, this study succeeded in obtaining specific strains of bacteria that are expected to be used in drug development and other applied research.

This study focused on *Waltera* spp., which have unique shapes and were isolated from several faecal samples. Although there are examples of new species obtained using agar medium as a method of isolation by co-culture [5], the growth of *Waltera* spp. isolates is not supported by co-culture on agar medium. Therefore, the growth of *Waltera* spp. isolates may be supported by SB in liquid-liquid co-culture rather than on agar media.

The cell diameters of *Waltera* spp. extracted with a pore size of 0.45 µm were smaller than 0.45 µm, suggesting that their passage through the 0.45 µm pore size filter was a selective pressure for their isolation. As shown in previous reports [11], *Waltera* spp. isolates, once obtained in co-culture, grew well in a monoculture in liquid medium and formed colonies on agar plates. However, the selected small-sized *Waltera* spp. isolates did not grow well in liquid medium in monocultures or formed colonies on agar plates but in co-culture. This suggests that the metabolites obtained by co-culture are necessary for the growth of selected small-sized *Waltera* spp. isolates. In contrast, the non-filtration strains grew better in monoculture than in co-culture. Organic acids, the major metabolites of the gut microbiome, are utilized by gut bacteria but have also been reported to inhibit the growth of gut bacteria [26,28]. This suggests that co-culture may be involved in the colony formation of selected small cells but may also inhibit their growth.

When the selected small-sized *Waltera* spp. isolates were incubated with the culture supernatant of the SB, the *Waltera* spp. isolates did not form colonies. Furthermore, no colonies formed when culture supernatants of the two isolated SB, *B. thetaiotaomicron* and *E. coli*, were added to the selected small *Waltera* spp. isolates. These results suggest that continuous co-culture of SB and *Waltera* spp. isolates is important for colony formation by *Waltera* spp. isolates, although the detailed mechanism remains unknown.

A few species, such as *B. thetaiotaomicron* and *E. coli*, have been isolated from SB under conditions in which *Waltera* spp. were isolated by co-culture. These limited species were not predominant in monocultures. Therefore, it is possible that the signals produced during co-culture supported an increase in *B. thetaiotaomicron* and *E. coli*. Several metabolites, such as indole, remained in the medium as candidates for the signal. Indole production has been observed in *B. thetaiotaomicron* and *E. coli* [29]. Indole has signalling effects, such as the inhibition of biofilm formation and the growth of certain bacteria [30], which may have inhibited the growth of miscellaneous bacteria. Furthermore, *E. coli* exhibits an avoidance response at indole concentration below 1 mM and a time-dependent shift to an attractant response at concentrations above 1 mM, suggesting that indole repels the invasion of foreign enemies due to differences in the adaptation state of bacteria, while at the same time attracting the growth of symbiotic bacteria adapted to indole [31]. Therefore, it is possible that the growth of indole-adapted bacterial species may have been induced in liquid-liquid co-culture. Indoles were also detected under co-culture conditions with *Bacteroides* spp. and *E. coli*. This finding reinforces the hypothesis that indole contributes to the growth of *Waltera* spp. Under these conditions, the concentrations of amino acids, some organic acids and nucleotides in the co-culture increased rapidly in the early stages of incubation and then gradually decreased. Because insufficient nutrition inhibits colony formation [32], these nutrients may be by *Waltera* spp. to form colonies. Therefore, some of these metabolites were added to the medium to culture selected small-sized *Waltera* spp. isolates. However, growth was not enhanced, and colony formation on the agar medium was not achieved. As described above, no growth of *Waltera* spp. was observed with the addition of the co-culture supernatant or some of the metabolites detected using metabolomic analysis. This indicates that a culture method in which mutual exchange occurs continuously is important for symbiosis between the SB and *Waltera* spp. Continuous culture may be necessary for bacterial growth [33], so the addition of culture medium alone may not support growth. For example, quorum-sensing signal molecules have been reported to correlate with cell resuscitation from viable but nonculturable (VBNC) conditions, suggesting that continuous bacterial conversations in co-culture may support growth of VBNC [34]. In addition, treatments such as sterilization to obtain the culture supernatant solution may increase the redox potential and hinder growth [35]. This suggests that continuous culture in the co-culture vessel used in this study may have contributed to the acquisition of the isolates. Alternatively, metabolites that were not detected using metabolomic analysis or were not added to the medium may have enhanced the growth of *Waltera* spp. Future clarification of the symbiotic relationship among *B. thetaiotaomicron*, *E. coli* and *Waltera* spp. will help to elucidate the growth-promoting factors of *Waltera* spp.

The liquid-liquid co-culture method used in this study helped us not only to isolate unique bacterial species but also to identify SB and understand metabolite variation. On the other hand, it is difficult to measure metabolites in real time in the co-culture system used in this study. A system that allows real-time measurement of metabolites detected during continuous co-culture would help to understand the growth of difficult-to-culture micro-organisms.

It is expected that liquid-liquid co-culture will lead to the isolation of various species other than those reported in this study. This co-culture method is not limited to the isolation of gut bacteria but can be applied to the isolation of micro-organisms in various environments. We expect that this co-culture method will become important for the isolation of new and difficult-to-culture micro-organisms in various environments and for elucidating symbiotic relationships.

## Supplementary material

10.1099/mic.0.001581 Uncited Supplementary Material 1.

## Abbreviations

BE: B. thetaiotaomicron 17EGH20 and Escherichia coli 17CBH1
JCMs: Japan Collection of Microorganisms
LC: liquid chromatography
RumA: Ruminococcin A
SB: supporting bacteria
VBNC: viable but nonculturable
