# Supplementary material for: Difficult-to-culture micro-organisms specifically isolated using the liquid-liquid co-culture method – towards the identification of bacterial species and metabolites supporting their growth
Source: Microbiology (Reading). 2025 Jul 8;171(7):001581. doi: 10.1099/mic.0.001581 (PMC12282286; doi:10.1099/mic.0.001581)
Supplement: Uncited Supplementary Material 1. [file mic-171-01581-s001.pdf]

# Difficult-to-culture micro-organisms specifically isolated by the liquid-liquid co-culture method -Towards the identification of bacterial species and metabolites supporting their growth-

Atsushi Hisatomi[1]*, Takanobu Yoshida[2], Tomohisa Hasunuma[2,3], Moriya Ohkuma[1] and Mitsuo Sakamoto[1,4]*

[1]Microbe Division/Japan Collection of Microorganisms, RIKEN BioResource Research Center, Tsukuba, Ibaraki 305-0074, Japan; [2]Engineering Biology Research Center, Kobe University, Nada, Kobe 657-8501, Japan; [3]Graduate School of Science, Technology and Innovation, Kobe University, Nada, Kobe 657-8501, Japan; [4]NODAI Culture Collection Center, Tokyo NODAI Research Institute, Tokyo University of Agriculture, Setagaya-ku, Tokyo 156-8502, Japan.

*Correspondence: Atsushi Hisatomi, atsushi.hisatomi@riken.jp; Mitsuo Sakamoto, sakamoto@riken.jp

**Supplementary Material - Tables S1, 2a, b**

**Table S1.** Compounds detected by metabolomic analysis added to YCFA medium

| Added compound | Concentration (μM) |
|---|---|
| Aspartic acid | 530 |
| Citric acid | 65 |
| Fumaric acid | 7 |
| Indol | 10 |
| Leucine | 3,880 |
| Lysine | 3,270 |
| Maleic acid | 260 |
| Malic acid | 13 |
| Nicotinamide | 18 |
| Phenylalanine | 1,160 |

When selected small-sized isolates are grown by co-cultivation,
the compound with a higher concentration in the early stages (3 h) of culture and a gradual decrease.

**Table S2a.** Metabolites increased when *Waltera acetigignens* 18YCFAH0.3Co2 is co-cultured with *Bacteroides thetaiotaomicron* 17EGH20 and *Escherichia coli* 17CBH1

| Co-culture time (h) | 2-Hydroxy-4-methylpentanoic acid | Cysteine |
|---|---|---|
| 0 | 1.44 | 4629.96 |
| 3 | 2.04 | 2341.98 |
| 6 | 2.09 | 2021.06 |
| 9 | 2.57 | 2652.04 |
| 12 | 5.90 | 2921.32 |
| 24 | 11.63 | 3302.95 |
| 36 | 23.96 | 3977.55 |
| 48 | 25.48 | 5161.44 |

The higher the production, the more it is shown in red.

**Table S2b.** Metabolites increased when *Waltera intestinalis* 19YCFAH0.3Co2 is co-cultured with *Bacteroides thetaiotaomicron* 17EGH20 and *Escherichia coli* 17CBH1

| Co-culture time (h) | 2-Aminobutyric acid | 2-Hydroxy-4-methylpentanoic acid | 2-Hydroxypentanoic acid | 4-Hydroxyphenylacetic acid | Cadaverine | Cystine | Glutamic acid | Lactic acid |
|---|---|---|---|---|---|---|---|---|
| 0 | 3.59 | 1.44 | 12583.02 | 0.08 | 2.96 | 85.67 | 3.41 | 1102.91 |
| 3 | 4.41 | 1.94 | 8399.81 | 0.16 | 21.61 | 22.52 | 1.00 | 1108.45 |
| 6 | 3.88 | 2.06 | 12211.67 | 0.14 | 7.20 | 24.00 | 4.04 | 1191.72 |
| 9 | 4.22 | 2.11 | 11196.01 | 0.16 | 58.95 | 33.86 | 3.65 | 1235.99 |
| 12 | 4.69 | 4.21 | 16670.86 | 0.35 | 142.63 | 43.58 | 4.67 | 1368.27 |
| 24 | 8.45 | 19.84 | 31485.10 | 8.89 | 348.86 | 61.55 | 15.68 | 1536.93 |
| 36 | 8.91 | 16.73 | 30223.64 | 9.73 | 345.40 | 60.33 | 15.39 | 1761.12 |
| 48 | 20.65 | 43.40 | 38707.57 | 26.33 | 330.42 | 78.83 | 29.24 | 1839.45 |

The higher the production, the more it is shown in red.
